# Action plan for drug-resistant Streptococcus pneumoniae. The DRSP Working Group.

**DOI:** 10.3201/eid0102.950208

**Published:** 1995

**Authors:** M S Cetron, D B Jernigan, R F Breiman


### Action Plan for Drug-Resistant *Streptococcus pneumoniae*

*Streptococcus pneumoniae* is a leading cause of illness and death in the United States. It accounts for an estimated 3,000 cases of meningitis, 50,000 cases of bacteremia, 500,000 cases of pneumonia, and more than seven million cases of otitis media annually (1, 2). *S. pneumoniae* had been almost uniformly susceptible to penicillin; however, with the development and worldwide spread of drug-resistant *S. pneumoniae* (DRSP), a public health challenge has emerged. Studies from Australia, Southeast Asia, Africa, and Europe have reported pneumococcal strains resistant to penicillin and other drugs (3). Surveillance data collected at the Centers for Disease Control and Prevention (CDC) have shown that high-level resistance to penicillin increased more than 60-fold—from 0.02% for 1979-1987 to 1.3% in 1992—for pneumococcal isolates from invasive infections (4). In some communities, at least 30% of isolates are nonsusceptible to penicillin (5; CDC, unpublished data).

Pneumococcal resistance has been reported for beta-lactams, macrolides, chloramphenicol, and sulfonamides. As multidrug-resistant strains become increasingly prevalent, treatment options will become limited. The clinical impact of antimicrobial resistance on the outcome of invasive and noninvasive DRSP infections remains largely unknown. Vancomycin has been required to treat patients with pneumococcal meningitis caused by strains resistant to extended-spectrum cephalosporins (e.g., cefotaxime and ceftriaxone) (6). Optimal treatment regimens for DRSP infections remain to be defined; CDC is organizing a working group to develop consensus guidelines for the management of pneumococcal infections.

The prevalence of pneumococcal resistance to antimicrobial drugs is not known for most areas of the United States since DRSP infection has not been a reportable condition. Some studies have suggested great geographic and temporal variation in levels of resistance; prevalence rates are 2% to 30% (5,7). In addition, DRSP can spread rapidly through a population, and the prevalence of resistant isolates can differ in adults and children. To make appropriate empiric antimicrobial choices, clinicians need a reliable and current assessment of the level of antimicrobial resistance in the community.

To address the growing problem of DRSP, a working group of public health practitioners, health care providers, clinical laboratorians, and representatives of key professional societies was formed in June 1994. The group identified the development of an electronic, laboratory-based surveillance system for DRSP as the essential first step to address this concern. The group has issued a comprehensive plan, "A National Strategy for the Surveillance, Applied Research, Control, and Prevention of DRSP," to be published in June 1995. This plan focuses on three public health priorities: 1) to define and monitor the prevalence and geographic distribution of DRSP and recognize the emergence of patterns of resistance, 2) to study further the epidemiology of DRSP, and 3) to minimize the complications of DRSP infections through control and prevention.

The working group has begun piloting an electronic, laboratory-based surveillance network to detect serious illness due to DRSP. For isolates nonsusceptible to oxacillin (zone size <20 mm), minimal inhibitory concentrations should routinely be determined for penicillin, an extended-spectrum cephalosporin, chloramphenicol, vancomycin, and other clinically relevant drugs. These data will be analyzed to determine community-specific levels of resistance and will be made available to clinicians to improve antimicrobial use. Additionally, aggregate data will be sent to CDC so that national trends in pneumococcal resistance can be identified and reported.

Clinical laboratory directors, large commercial laboratory operators, and laboratory software manufacturers indicate that many laboratory software systems can accommodate a paperless, automated mechanism for reporting communicable disease information directly to public health authorities. DRSP surveillance may thus serve as a model for electronic, laboratory-based reporting for other laboratory-reportable conditions. Although improved data flow should increase the number and timeliness of reported cases, a strategy for ensuring quality control of data will be required.

In the era of emerging antimicrobial resistance, prevention of pneumococcal infections is paramount; vaccination strategies offer an important approach to controlling DRSP. An existing pneumococcal polysaccharide vaccine that can prevent a substantial number of pneumococcal infections, including those caused by DRSP, is underutilized. The vaccine is recommended by the Advisory Committee for Immunization Practices (ACIP) for use in persons older than 2 years of age who have certain underlying medical conditions and for all persons older than 65 years of age (8). It is not recommended for routine use among children under 2 years of age because it does not provide immunity consistently in this age group. An effective vaccine is needed to prevent pneumococcal infections in this population, which has the highest risk for otitis media and meningitis caused by DRSP. If the prevalence of pneumococcal infection (and therefore antimicrobial use) can be substantially reduced by vaccination, the impact of DRSP may diminish. Novel vaccine demonstration projects supported by federal and state health agencies are under way to explore means of increasing coverage with the effective 23-valent pneumococcal polysaccharide vaccine.

Applied research is also needed to address the problem of DRSP. CDC has recently funded population-based investigations to define risk factors, patterns of transmission, costs, and health outcomes associated with DRSP. Public health programs for control and prevention of DRSP are being designed for use at local, state, and federal levels. Because unnecessary use of antimicrobial agents has contributed to the emergence of resistant bacteria (1, 3, 5), educational materials and campaigns are being developed for both health care providers and consumers to raise awareness of the link between excessive antimicrobial use and the emergence of drug-resistant organisms. Through a multifaceted approach, the growing problem of DRSP can be addressed to minimize the complications and costs of resistant pneumococcal infections. Surveillance for DRSP is an important starting point from which control and prevention solutions can proceed.

For more information regarding DRSP activities at CDC, write to Division of Bacterial and Mycotic Diseases, NCID, CDC Mailstop C-09, 1600 Clifton Rd., Atlanta, GA 30333 or send an e-mail to DRSP@ciddbd1.em.cdc.gov.

**Martin S. Cetron, Daniel B. Jernigan, Robert F. Breiman, and the DRSP Working Group***
National Center for Infectious Diseases
Centers for Disease Control and Prevention
Atlanta, Georgia, USA

* Guthrie Birkhead, New York State Department of Health and the Council of State and Territorial Epidemiologists (CSTE); Jay C. Butler, CDC; Mathew L. Cartter, Connecticut Department of Public Health and Addiction Services; Joan P. Chesney, American Academy of Pediatrics; William Craig, Infectious Diseases Society of America; Robert P. Gaynes, CDC; Mary J. R. Gilchrist, American Society of Microbiologists; Richard E. Hoffman, Colorado Department of Public Health and Environment and CSTE; James Jorgensen, National Committee for Clinical Laboratory Standards; David Klein, National Institute of Allergy and Infectious Diseases, National Institutes of Health; Thomas O'Brien, World Health Organization Collaborating Center for Antibiotic Resistance, Boston; Benjamin Schwartz, CDC; Albert Sheldon, Jr., Food and Drug Administration; Kenneth C. Spitalny, New Jersey State Department of Health; Fred C. Tenover, CDC; and Ralph J. Timperi, Association of State and Territorial Public Health Laboratory Directors.

### References

1. Reichler MR, Allphin AA, Breiman RF, et al. The spread of multiply resistant *Streptococcus pneumoniae* at a day care center in Ohio. J Infect Dis 1992;166:1346-53.
2. Stool SE, Field MJ. The impact of otitis media. Pediatr Infect Dis J 1989;8:S11-S14.
3. Klugman KP. Pneumococcal resistance to antibiotics. Clin Microbiol Rev 1990;3:171-96.
4. Breiman RF, Butler JC, Tenover FC, Elliott JA, Facklam RR. Emergence of drug-resistant pneumococcal infections in the United States. JAMA 1994;271:1831-5.
5. Centers for Disease Control and Prevention. Drug-resistant *Streptococcus pneumoniae*—Kentucky and Tennessee, 1993. MMWR 1994;43:23-5,31.
6. Leggiadro RJ, Barrett, FF, Chesney PJ, Davis Y, Tenover FC. Invasive pneumococci with high level penicillin and cephalosporin resistance at a mid-South children's hospital. Pediatr Infect Dis J 1994;13:320-2.
7. Centers for Disease Control and Prevention. Prevalence of penicillin-resistant *Streptococcal pneumoniae*—Connecticut 1992-1993. MMWR 1994;43:216-7, 223.
8. Advisory Committee for Immunization Practices. Update on adult immunization: recommendations of the Immunization Practices Advisory Committee (ACIP). MMWR 1991;40 (RR-12):42-4.

